# Editorial: The AMPD in Clinical and Applied Practice: Emerging Trends and Empirical Support

**DOI:** 10.3389/fpsyg.2022.867933

**Published:** 2022-03-17

**Authors:** Mark H. Waugh, Abby L. Mulay, Gina Rossi, Kevin B. Meehan

**Affiliations:** ^1^Health Services Division, Oak Ridge National Laboratory, Oak Ridge, TN, United States; ^2^Department of Psychology, University of Tennessee, Knoxville, TN, United States; ^3^Community and Public Safety Psychiatry Division, Department of Psychiatry and Behavioral Sciences, Medical University of South Carolina, Charleston, SC, United States; ^4^Personality and Psychopathology Research Group, Department of Psychology, Vrije Universiteit Brussel (VUB), Brussels, Belgium; ^5^Department of Psychology, Long Island University – Brooklyn, New York, NY, United States

**Keywords:** DSM-5 AMPD, personality disorder, alternative model for personality disorders, ICD-11 personality disorder, dimensional model of personality disorder

The maxim “There is nothing so practical as a good theory” is attributed to field theorist Kurt Lewin. This maxim applies to the DSM-5 Alternative Model for Personality Disorders (AMPD) and the ICD-11 dimensional model for personality disorders. Both models derive from major theoretical traditions in personality and psychopathology (Waugh et al., 2017; Hopwood et al., 2019; Bach et al., 2021) and offer a practical approach to operationalize personality disorder (PD) diagnosis, incorporating dimensional rather than categorical representation of PD.

The AMPD was published in 2013 in the DSM-5. Advance work with the model appeared even earlier (e.g., Bender et al., 2011). Now about 10 years out, the vanguard instantiation of dimensional representation in diagnostic nosology that is the AMPD, it has achieved a degree of maturity. Google Scholar lists 267,000 citations (accessed January 30, 2022). Reviews of the AMPD such as by Hopwood et al. (2019); Rodriguez-Seijas et al. (2019); Zimmermann et al. (2019); Krueger and Hobbs (2020), or Birkhölzer et al. (2021) can bring the interested reader up to date. This current Research Topic of Frontiers of Psychology, “*The AMPD in Clinical and Applied Practice: Emerging Trends and Empirical Support*,” adds new and needed applied studies illustrating the AMPD in clinical practice.

Two articles creatively feature historically important classic case examples to examine practical aspects of the AMPD. Drawing on the interpersonal perspective embedded within the AMPD and innovatively applying idiographic ecological momentary assessment methods, Luo et al. identified specific dynamics associated with psychotherapy alliance ruptures within the treatment of the classic case of “Gloria” by Carl Rogers, Fritz Perls, and Albert Ellis. Using the classic case of “Madeline,” who was evaluated by major figures in personality assessment twice over nearly 20 years, Garner et al. studied beginning clinicians learning and applying both the AMPD and the Shedler-Westen Assessment Procedure (SWAP; Shedler and Westen, 2007). They found, consistent with previous studies, the AMPD is very learnable, including the Level of Personality Functioning Scale (LPFS). However, they also reveal that beginning clinicians may be inclined to underestimate severity in LPFS ratings, an important point which may inform clinical training with the AMPD.

Patients with PD often participate in psychotherapy more than once and with more than one therapist. Bliton et al. studied this common clinical situation using two patients seen over three years by different clinicians. Assessing both PD severity (LPFS; Criterion A) and style of maladaptive personality traits (Criterion B), they found psychotherapeutic change manifested in decreased severity of PD while style of maladaptive traits was more stable. Their results are theoretically important and further emphasize the relevance of assessing LPF in treatment planning. Similarly, Riegel et al. introduced the AMPD to an important clinical application: pre-bariatric surgery psychological evaluation. Characterizing presurgical and general psychiatric patients as well as non-clinical respondents, they found meaningful groups defined by measures of Criterion A and B. Their results suggest the maladaptive trait domains of Negative Affectivity and Detachment are important domains to evaluate in bariatric pre-surgical assessments. These findings also point to the relevance of the AMPD in behavioral medicine contexts.

The multiple versions of the Minnesota Multiphasic Personality Inventory (MMPI) family of instruments are widely used clinical assessment tools. Waugh et al. studied the influence of Criterion A in existing PD syndrome scales scored from MMPI responses. They found that MMPI-2 and MMPI-2-RF PD scales include meaningful LPF variance beyond that accounted for by maladaptive traits and demoralization or general distress. These results not only contribute to cross walking the AMPD and the MMPI family of instruments but also show that LPF is more than maladaptive trait severity, in part echoing the findings of Bliton et al.

Each of these articles speaks to interests important to the clinician by demonstrating the clinical usefulness of the AMPD. Moreover, studies of practical applications of the AMPD further the construct validation of the AMPD approach and dimensional diagnosis of PD in general. As well, studies such as featured in this special section of *Frontiers of Psychology* provide clinicians with important knowledge on how to apply AMPD measures in the detection of personality pathology in clinical practice. The theme of clinical relevance uniting these articles echoes Bender's (2019) call to remember the “importance of being human”—that is, staying experience near–in our conceptualization of psychopathology. Three of the five articles employ idiographic assessment, an approach of great relevance to clinicians. Furthermore, narrative identity and meaning-making are relatively under-emphasized in the study of dimensional models of PD (Lind et al., 2020, 2022). Idiographic study and application of the AMPD provides much needed attention to this aspect of personhood. We believe the reader will find interest and value within this Research Topic and that Kurt Lewin was correct about the practicality of theory. A close reading of these papers may advance the treatment of persons suffering from PDs.

## Author Contributions

All authors listed have made a substantial, direct, and intellectual contribution to the work and approved it for publication.

## Licenses and Permissions

This manuscript has been authored by UT-Battelle, LLC, under contract DE-AC05-00OR22725 with the US Department of Energy (DOE). The United States Government retains and the publisher, by accepting the article for publication, acknowledges that the United States Government retains a non-exclusive, paid up, irrevocable, world-wide license to publish, or reproduce the published form of this manuscript, or allow others to do so, for United States Government purposes. The Department of Energy will provide public access to these results of federally sponsored research in accordance with the DOE Public Access Plan (http://energy.gov/downloads/doe-public-access-plan).

## Author Disclaimer

This work represents the opinion MW and not of the U.S. DOE or ORNL.

## Conflict of Interest

The authors declare that the research was conducted in the absence of any commercial or financial relationships that could be construed as a potential conflict of interest.

## Publisher's Note

All claims expressed in this article are solely those of the authors and do not necessarily represent those of their affiliated organizations, or those of the publisher, the editors and the reviewers. Any product that may be evaluated in this article, or claim that may be made by its manufacturer, is not guaranteed or endorsed by the publisher.
